# Cryptococcal Meningitis Causing Refractory Hemichorea-Hemiballismus Treated With Pallidotomy

**DOI:** 10.7759/cureus.16493

**Published:** 2021-07-19

**Authors:** Kevin G Buell, Brian P Vickers, Karen C Bloch, Amy E Brown, Peter Hedera, Walter Jermakowicz, Peter E Konrad, E Wesley Ely

**Affiliations:** 1 Department of Medicine, Vanderbilt University Medical Center, Nashville, USA; 2 Division of Infectious Diseases, Vanderbilt University Medical Center, Nashville, USA; 3 Department of Neurology, Vanderbilt University Medical Center, Nashville, USA; 4 Department of Neurology, University of Louisville, Louisville, USA; 5 Department of Neurosurgery, Vanderbilt University Medical Center, Nashville, USA; 6 Critical Illness, Brain Dysfunction, and Survivorship (CIBS) Center; and Division of Allergy, Pulmonary, and Critical Care Medicine, Vanderbilt University Medical Center, Nashville, USA; 7 Geriatric Research Education Clinical Center, Tennessee Valley Veteran’s Affairs, Nashville, USA

**Keywords:** cryptococcal meningitis, cryptococcus neoformans, hemichorea, hemiballismus, pallidotomy

## Abstract

We report a case of a 31-year-old immunocompetent male who presented with altered mental status and agitation requiring intubation. As sedation was weaned, he demonstrated choreiform movements with associated hemiballismus of the right upper and lower extremities, and he was ultimately diagnosed with cryptococcal meningitis. The patient’s chorea did not terminate after the completion of induction antifungal therapy and all pharmacologic options for the management of chorea were ineffective. He underwent a successful unilateral pallidotomy using standard stereotactic methodology targeting the posterior-ventral pallidum, and his choreiform movements dramatically improved post-operatively within 48 hours.

## Introduction

Chorea is a movement disorder characterized by irregular, flowing, random, involuntary movements. Ballism is related to chorea and is characterized by large-amplitude movements that are jerking or flailing in appearance, and many times are superimposed on choreiform movements [1]. Focal chorea-ballism can be triggered by infectious, metabolic, hormonal, vascular, or drug-induced insults to the basal ganglia. Secondary chorea from infection is typically caused by structural changes in the basal ganglia. Although previously described in case reports of viral encephalitis, neurosyphilis, cerebral toxoplasmosis, tuberculous and bacterial meningitis [2], cryptococcal meningitis causing chorea is rare [3,4]. We present a case of a patient with cryptococcal meningitis causing hemichorea-hemiballismus refractory to pharmacotherapy who underwent a successful ablative surgical pallidotomy.

## Case presentation

A 31-year-old incarcerated male with schizophrenia and untreated chronic hepatitis C was transferred to our facility for the evaluation of altered mental status and agitation. During the week preceding admission, the penitentiary staff reported that he was uncharacteristically digging through trash cans and had become increasingly agitated. Upon transfer to our hospital, the patient was already intubated and deeply sedated. As sedation was weaned, he demonstrated repetitive, involuntary, non-fatigable choreiform movements with associated hemiballismus of the right upper and lower extremities (Video 1).

**Video 1:** Hemichorea-hemiballismus The video illustrating the patient’s persistent hemichorea-hemiballismus despite treatment with high dose tetrabenazine, haloperidol, olanzapine, lorazepam, diphenhydramine, clonidine, and guanfacine.

**Figure 1:**
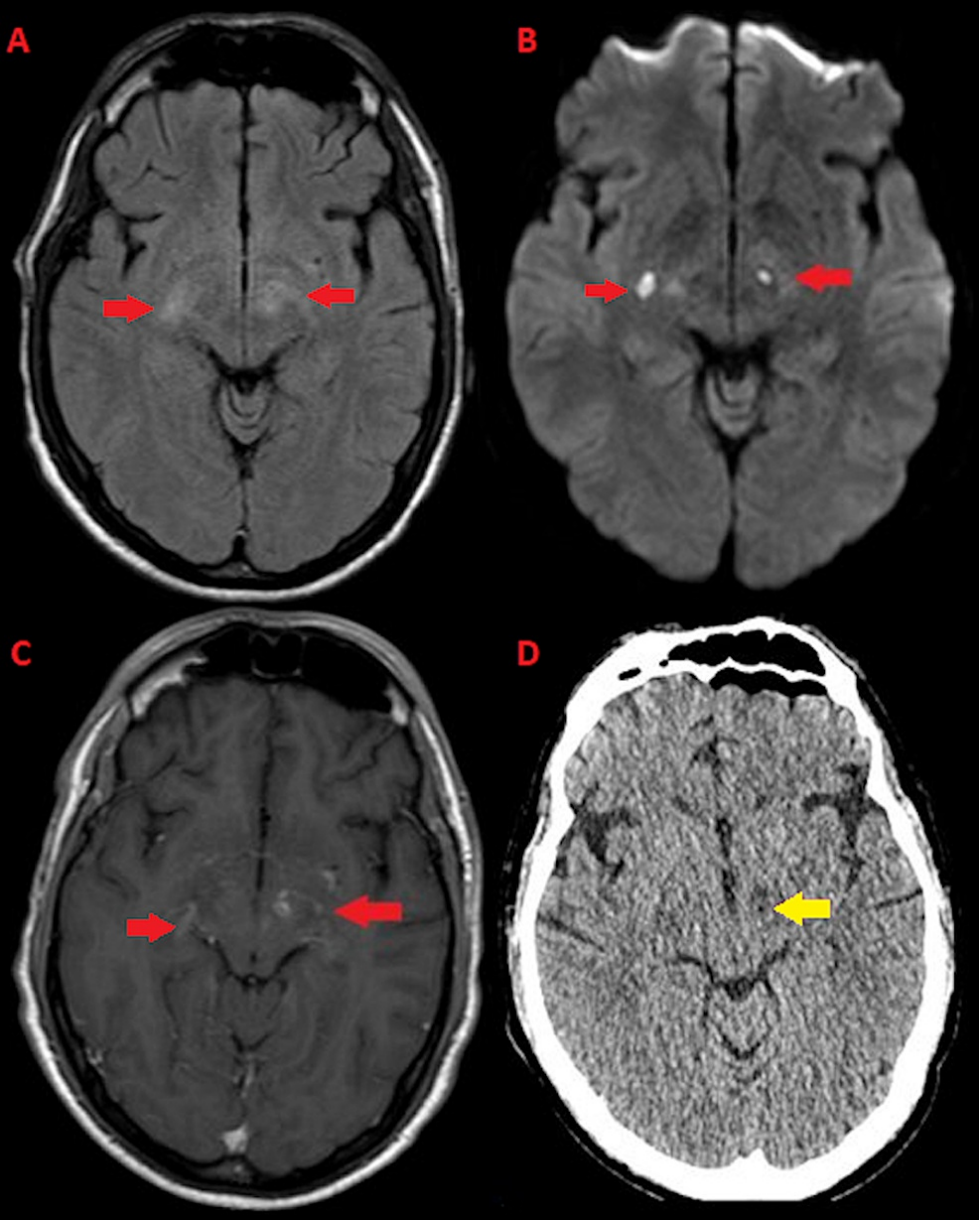
Magnetic resonance imaging of the brain Brain MRI demonstrating abnormal T2-weighted-fluid-attenuated inversion recovery hyperintense signal (a) and restricted diffusion (b) with associated enhancement (c) in the globus pallidus and internal capsule, suggestive of bilateral abscesses (red arrows). Post-surgical CT (d) with evidence of successful left pallidotomy (yellow arrow).

He did not have any focal weakness or any other focal neurological signs on examination. Relevant findings on laboratory evaluation were white blood cells 17.2 x 10^9^/L (3.5-10.5 x 10^9^/L) and creatine phosphokinase 6950 unit/L (30-200 unit/L). The remainder of his complete blood count, basic metabolic, hepatic, and coagulation profiles were unremarkable. HIV testing was negative and the hepatitis C viral load was 153,767 IU/ml. Urine drug screen was positive for benzodiazepines only. MRI of the brain demonstrated abnormal restricted diffusion and T2-weighted-fluid-attenuated inversion recovery (T2-FLAIR) hyperintense signal with associated enhancement in the globus pallidus, internal capsule, and cerebral peduncles bilaterally, representing multiple small areas of cerebritis and abscess formation within the basal ganglia (Figures 1A-1D). A lumbar puncture was performed and had an opening pressure of 27 centimeters of water with 36 red blood cells/microliter, 89 nucleated cells/microliter (69% lymphocytes, 15% neutrophils, and 11% monocytes), 32 mg/dL of glucose (45-75 mg/dL), and 118 mg/dL of protein (15-50 mg/dL) in the cerebrospinal fluid (CSF). The CSF cryptococcal antigen was positive with a 1:320 titer and fungal culture grew *Cryptococcus neoformans* (grubii subtype by molecular typing). The patient was initiated on intravenous amphotericin, dexamethasone, and oral flucytosine. 

Despite successful extubation, the patient remained confined to the intensive care unit for over 50 days due to the need for analgosedation to control the violent flailing of his leg. Tetrabenazine, haloperidol, olanzapine, lorazepam, amantadine, valproic acid, and gabapentin were unsuccessfully titrated to the maximal tolerated dose. He underwent six lumbar punctures for the management of raised intracranial pressure. The CSF protein and glucose both normalized on hospital day 15 and CSF pleocytosis declined to 1 white blood cell/mcL on hospital day 26. His cryptococcus antigen titers sequentially decreased to a final titer of 1:10 and the last opening pressure was recorded at 13 centimeters of water. Repeat MRI on hospital day 30 showed improvement in restrictive diffusion, T2-FLAIR hyperintensities, and resolution of the enhancement in the basal ganglia and cerebellum.

After completing six weeks of induction antifungal therapy, the patient’s distressing hemichorea-hemiballismus was not controlled despite normalization of CSF parameters and radiologic improvement noted on repeat MRI. As the pharmacologic options had been exhausted for his movement disorder, he underwent a unilateral left pallidotomy on hospital day 56. Using standard stereotactic methodology, the posterior-ventral pallidum was targeted and intraoperative test stimulation was used to avoid capsular lesioning. Three lesions (60 seconds at 80°C) were delivered to posteroventral globus pallidus interna (anterior commissure-posterior commissure {AC-PC}: anterior-posterior {AP} = +2.1 mm, mediolateral {ML} = -19.0 mm, superior-inferior (SI) = -3.2, -6.2, -9.2mm). His choreiform movements dramatically improved within 48 hours, and he was discharged from the hospital. Six weeks later, he was able to walk during his follow-up appointment and his hemichorea-hemiballismus had not recurred (Video 2).

**Video 2:** Post unilateral left pallidotomy The video illustrating the patient walking without choreiform movements during a follow-up outpatient appointment six weeks after his surgery.

## Discussion

Cryptococcus neoformans is an environmental fungus that is inhaled and can disseminate to the central nervous system via hematogenous spread. Approximately 20% of central nervous system infections are seen in HIV-negative patients, of which 10-40% have no apparent immune deficiency [5,6]. Risk factors for cryptococcal meningitis in HIV-negative patients include decompensated liver disease, sarcoidosis, systemic lupus erythematosus, solid organ transplantation, hematological malignancy, and cell-mediated immunosuppression by medications such as steroids [7].

Cryptococcal meningitis presents over one-to-two weeks with headache, altered mental status, and behavioral changes. Less than 15% of patients will have detectable cranial nerve palsies, pathologic reflexes, papilledema, or signs of cerebellar deficits at presentation. In HIV-negative patients, the median values on CSF analysis have been reported as 73 leukocytes/mm^3^, 7 erythrocytes/mm^3^, 100 mg/dL protein, and 42 mg/dL glucose. The sensitivity of CSF culture is approximately 90% regardless of HIV status [5]. MRI can be normal, but in three-quarters of cases, will demonstrate temporal lobe and basal ganglia leptomeningeal enhancement in a micronodular pattern, gelatinous pseudocysts, and abscess formation [8].

The treatment of cryptococcal meningitis is divided into three phases: induction, consolidation, and maintenance. Induction therapy is comprised of amphotericin and flucytosine. In HIV-negative patients, the presence of neurologic complications warrants an extended induction therapy of six weeks compared to four weeks in patients without neurologic manifestations, who successfully clear CSF cultures on repeat lumbar puncture. Consolidation therapy with fluconazole follows for eight weeks and is subsequently dose reduced for a further six to 12 months in the maintenance phase. Despite treatment, cryptococcal meningitis can cause severe neurologic sequelae like intracerebral edema, hydrocephalus, seizures, intellectual disability, and focal neurologic deficits. 

The management of acquired chorea and ballism can be challenging. As choreiform movements result from excessive dopaminergic activity and loss of inhibition on hyperkinetic movements, medications suppressing the effect of the dopaminergic axis can be used to control symptoms while the underlying trigger for acquired chorea is reversed [1]. Tetrabenazine, a reversible dopamine-depleting drug that inhibits pre-synaptic monoamine transportation, was the first drug approved by the United States Food and Drug Administration in the treatment of Huntington’s disease. Deutetrabenazine was subsequently approved and has the additional benefit of a lower side effect profile. Off-label use of first- and second-generation antipsychotics has been employed for their dopamine receptor blocking properties [9]. In our patient, both were trialed to the highest tolerated doses but were unfortunately ineffective. 

Invasive treatments, including targeting globus pallidus pars interna (GPi) have been used in medically resistant cases of chorea and ballism [10]. Surgical therapy may include deep brain stimulation (DBS) or lesioning procedures. Therapeutic recommendations are currently based on expert opinion, case reports, and small open-label studies. Both techniques have their respective advantages and disadvantages and there is an urgent need for larger, prospective, and multicenter studies. In the meantime, all treatment plans should be tailored to the individual patient using a multidisciplinary approach with local expertise. We selected lesioning surgery because the patient only required a unilateral procedure and the expected benefits tend to emerge faster. Furthermore, there are no potential DBS hardware infectious complications in patients with transmissible causes of chorea. Our experience also supports targeting GPi in patients with abnormal movements caused by structural changes in basal ganglia. Disruption of outflow from the sensorimotor region of GPi, localized in the posteroventral pallidum is effective in controlling abnormal movements and surgical results tend to be superior to targeting ventrolateral thalamus [10].

Even though surgical therapies in secondary chorea-ballism are less well established, our case further supports that stereotactic radiofrequency pallidotomy is an effective treatment of hemichorea-hemiballismus caused by cryptococcal meningitis and, in general, remains an attractive surgical option for medication-resistant hemiballismus patients [11].

## Conclusions

Cryptococcal meningitis can cause devastating neurologic deficits in immunocompetent patients despite appropriate treatment. Secondary chorea can be a rare complication of cryptococcal meningitis. For patients with choreiform movements that persist after the completion of induction antifungal therapy, stereotactic radiofrequency pallidotomy can be an attractive surgical option for medication-resistant hemiballismus.
